# Synergistic Effect of Simultaneous Doping of Ceria Nanorods with Cu and Cr on CO Oxidation and NO Reduction

**DOI:** 10.1002/chem.202004623

**Published:** 2020-12-27

**Authors:** Shawn C. Rood, Oriol Pastor‐Algaba, Albert Tosca‐Princep, Bruno Pinho, Mark Isaacs, Laura Torrente‐Murciano, Salvador Eslava

**Affiliations:** ^1^ Centre for Sustainable Chemical Technologies Department of Chemical Engineering University of Bath Claverton Down Bath BA2 7AY UK; ^2^ Departament d'Enginyeria Química, Biològica i Ambiental Universitat Autònoma de Barcelona Bellaterra 08193 Spain; ^3^ Department of Chemical Engineering and Biotechnology University of Cambridge Philippa Fawcett Drive Cambridge CB3 0AS UK; ^4^ Department of Chemistry University College London London WC1H 0AJ UK; ^5^ Department of Chemical Engineering Imperial College London London SW7 2AZ UK

**Keywords:** cerium, CO oxidation, hydrothermal synthesis, nanorods, NO reduction

## Abstract

Ceria particles play a key role in catalytic applications such as automotive three‐way catalytic systems in which toxic CO and NO are oxidized and reduced to safe CO_2_ and N_2_, respectively. In this work, we explore the incorporation of Cu and Cr metals as dopants in the crystal structure of ceria nanorods prepared by a single‐step hydrothermal synthesis. XRD, Raman and XPS confirm the incorporation of Cu and Cr in the ceria crystal lattices, offering ceria nanorods with a higher concentration of oxygen vacancies. XPS also confirms the presence of Cr and Cu surface species. H_2_‐TPR and XPS analysis show that the simultaneous Cu and Cr co‐doping results in a catalyst with a higher surface Cu concentration and a much‐enhanced surface reducibility, in comparison with either undoped or singly doped (Cu or Cr) ceria nanorods. While single Cu doping enhances catalytic CO oxidation and Cr doping improves catalytic NO reduction, co‐doping with both Cu and Cr enhances the benefits of both dopants in a synergistic manner employing roughly a quarter of dopant weight.

## Graphical abstract

**Doped up for detox**: Ceria particles play a key role in catalytic applications such as automotive three‐way catalytic systems in which toxic CO and NO are respectively oxidized and reduced to safe CO_2_ and N_2_. We explored the incorporation of Cu and Cr metals as dopants in the crystal structure of ceria nanorods prepared by a single‐step hydrothermal synthesis. Such co‐doped ceria nanorods show synergistic improvement in reducibility and enhanced performance for CO oxidation and NO reduction.

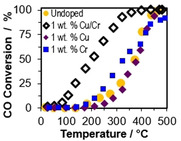

## Introduction

Ceria is widely used in catalytic applications across a broad range of fields, such as automotive three‐way catalysis, due to its oxygen storage capability.[^1^, ^2^, ^3^, ^4^, ^5^, ^6^, ^7^, ^8^] Doping ceria by replacing cerium atoms with other readily available metals has the potential to improve their low‐temperature performance, otherwise typically achieved by the addition of expensive Pt‐group metals.[^9^, ^10^, ^11^] In particular, the replacement of cerium atoms with other elements, particularly those of lower valence, can lead to the creation of additional oxygen vacancies within the lattice structure to compensate for the charge difference, improving redox capabilities.^[12]^ Additionally, the presence of elements with an ionic radius very different from that of cerium can create lattice defects and distortions, another contributor to oxygen vacancy concentration.^[9]^ Co‐doping ceria with multiple elements has been recently investigated for various applications. With regard to CO oxidation, DFT experiments show promising results for Mn/Fe‐doped ceria, and experimental results have been reported for Mn/Cu and Ag/Cu‐doped ceria.[^13^, ^14^, ^15^, ^16^] A co‐doped Mn/Co‐ceria catalyst has also been reported with enhanced performance for the reduction of NO by CO.^[17]^ On the other hand, not all dopant combinations provide better catalytic properties compared with similarly prepared singly doped ceria materials, demonstrating the need to carefully consider dopant properties and their effect on catalytic activity.^[18]^

While the suitability of doped ceria for CO oxidation has been widely examined, investigation into NO_x_ reduction has been less exhaustive.[^19^, ^20^, ^21^, ^22^, ^23^, ^24^, ^25^, ^26^, ^27^] While doping of ceria is generally a good strategy for catalytic oxidation reactions because it improves reducibility and oxygen vacancy formation, this can lead to a trade‐off of reducing the favorability of oxygen vacancy healing, necessary to complete the catalytic cycle for NO_x_ reduction.[^28^, ^29^] For this reason, both reactions must be examined simultaneously.

In this work, a template‐free hydrothermal method is used to synthesize Cu and Cr‐doped ceria nanorods with improved activities as low‐temperature automotive catalysts. Ceria nanorods are chosen for their preferential exposure of the (110) ceria facet that contains more surface oxygen vacancies and offers higher catalytic activity than other crystal facets.[^30^, ^31^, ^32^] The doped ceria nanorods are tested as catalysts for both CO oxidation and NO reduction. Due to improved reducibility, it is shown that doping ceria with Cu improves CO oxidation conversions, but is not so helpful for the reduction of NO. Conversely, doping ceria with Cr does not improve the CO oxidation conversions but does result in a catalyst with better low‐temperature performance for NO reduction. Co‐doping ceria with both Cu and Cr provides a catalyst with enhanced performance for both CO oxidation and NO reduction. Co‐doping synergistic effects moreover significantly decrease the amount of doping needed for an enhanced performance. The use of doped ceria nanorods offers an attractive route to producing automotive catalysts with better low‐temperature performance.

## Results and Discussion

**Figure 1:**
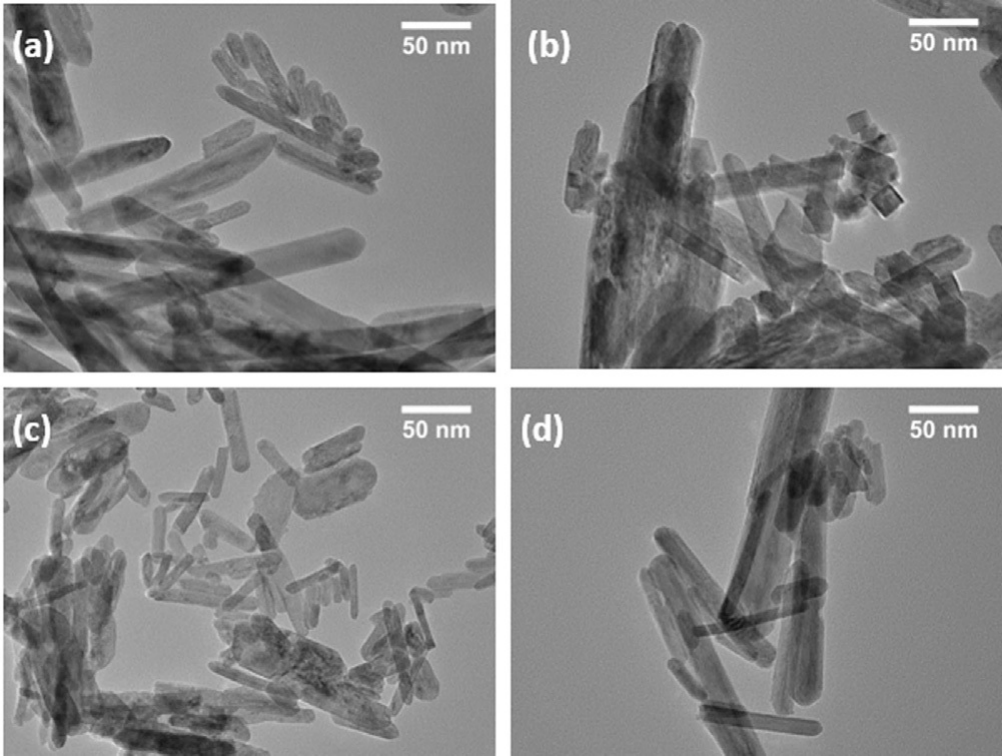
Representative TEM images of (a) undoped ceria nanorods, (b) 7 % Cu‐ceria nanorods, (c) 5 % Cr‐ceria nanorods, and (d) 1 % Cu/1 % Cr‐ceria nanorods.

Undoped and doped ceria nanorods were hydrothermally synthesized, filtered and washed, and dried under vacuum. The undoped ceria synthesis produced a pale‐yellow powder product, while Cu and/or Cr‐doped ceria produced varying shades of brown. The co‐doped Cu/Cr‐ceria sample was synthesized with a 1 % loading of each dopant for ease of comparison with singly doped ceria samples with equivalent or higher levels of Cu or Cr. Transmission electron microscopy (TEM) representative micrographs of undoped ceria, 7 % Cu‐ceria, 5 % Cr‐ceria, and 1 % Cu/1 % Cr‐ceria are shown in Figure 1. In all cases, there is a range of nanorod sizes: with nanorod widths generally below 50 nm and nanorod lengths ranging from 100 to several hundred nm. In the Cu‐doped ceria sample, a minority of small nanoparticles and nanocubes can be seen in addition to nanorods. In all cases, there is no evidence of formation of Cu and/or Cr particles outside the rod structure at these levels of doping below 7 %.

**Figure 2:**
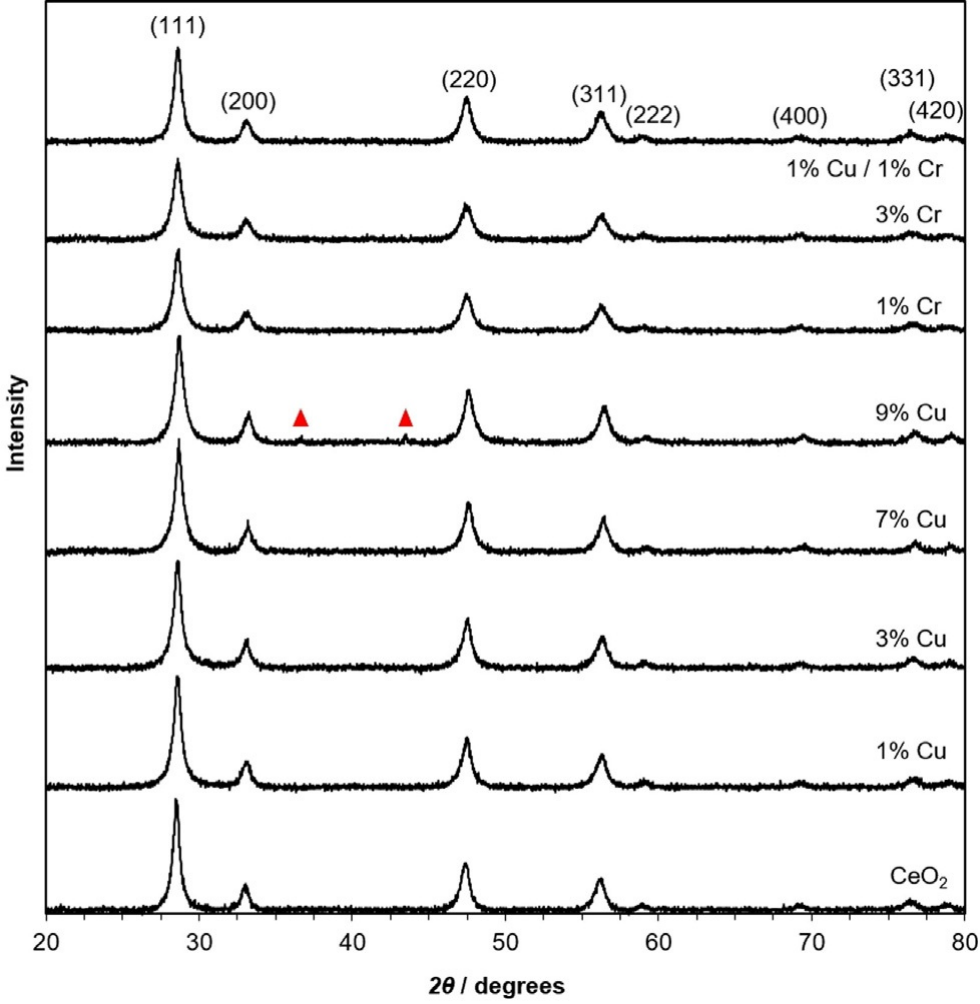
Powder XRD patterns of undoped, Cu‐doped, and Cr‐doped ceria nanorods. Ceria diffraction peaks are labelled with their Miller indices; ▴ represents copper oxide diffraction peaks.

Powder X‐ray diffraction (XRD) patterns of doped and undoped ceria nanorods are shown in Figure 2. The main diffraction peaks of fluorite‐type ceria are observed in all the samples (JCPDS 34‐0394). For copper‐doped ceria between 1 and 7 % Cu and chromium‐doped ceria at all dopant loadings synthesized (1 to 9 % Cr), only ceria diffraction peaks are seen. While this is expected for the 1 % Cu‐ceria due to a detection limit of approximately 1 %, for the samples with higher copper levels, this is an indication of dopant substitution into the ceria lattice and formation of a homogeneous fluorite structure.^[33]^ For the 1 % Cu/1 % Cr‐ceria, only ceria can be identified in the diffraction pattern as well. Anyway, the exclusive diffraction of ceria does not eliminate the possibility of highly dispersed copper oxide or chromium oxide species on the surface of the ceria. For 9 % Cu‐ceria, two additional diffraction peaks are seen at 36.9 and 43.5° 2*θ*—likely Cu_2_O.^[34]^ Therefore, it is likely that at loadings below 7 % Cu, copper atoms are introduced within the ceria lattice, substituting cerium with copper ions, but above 7 %, additional copper atoms are unable to be substituted into the lattice and agglomerate into copper oxide particles.

**Figure 3:**
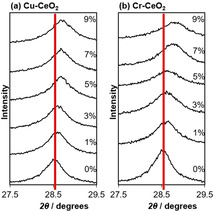
(111) Diffraction peak shift for (a) Cu‐doped and (b) Cr‐doped ceria nanorods prepared with different dopant concentrations.

**Figure 4:**
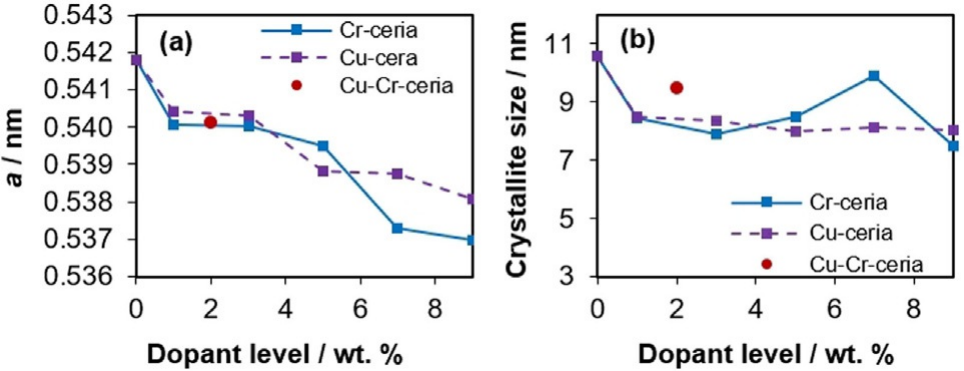
(a) Unit cell parameter ′a′ vs. dopant wt % and (b) coherent diffraction domain sizes (crystallite size) vs. dopant wt % for Cu and Cr‐doped ceria.

A small shift to higher 2*θ* values can be seen in the diffraction patterns with both dopants, shown in Figure 3. For Cu‐ceria and Cr‐ceria, a higher level of dopant corresponds to a larger peak shift, indicative of increasing levels of incorporation into the crystal lattice.^[33]^ In the case of copper, this shift only continues up to 7 % Cu; at higher copper dopant levels, there is no further shift which further support the formation of external copper oxide particles at higher loading levels. The Ce^4+^ ionic radius is 0.97 Å and Ce^3+^ is 1.14 Å, compared with for example, 0.73 Å for Cu^2+^ and 0.44 Å for Cr^6+^.^[35]^ Therefore, it is expected that increasing levels of dopant will decrease the unit cell size if the dopant is successfully integrated into the lattice structure. Correspondingly, unit cell values calculated using Bragg's Law for the doped ceria materials show a decrease in the size of ceria's unit cell with increasing levels of dopant (Figure 4 a). These results indicate that the Cu and Cr dopants substitute Ce ions in the crystal structure rather than occupy interstitial or surface sites. Anyway, as explained before, the substitution of Ce ions in the crystal structure does not eliminate the possibility of highly dispersed copper oxide or chromium oxide species on the surface of the ceria.

In addition to unit cell dimensions, coherent diffraction domain (crystallite) size calculations using the Scherrer equation show that increasing the amount of dopant decreases the domain size, shown in Figure 4 b. This is potentially related to the preparation method used for the nanorod synthesis. When the cerium precursor is added to the NaOH solution, Ce(OH)_3_ nuclei are formed. During hydrothermal synthesis, these nuclei dissolve and then recrystallize into nanorods, growing anisotropically.^[36]^ High levels of additional ions in the solution can interfere with this process, which would explain the nanoparticles and small nanocubes seen in Figure 1 b in addition to ceria nanorods. However, Cr‐doped ceria does not follow this trend of decreasing crystallite size past a dopant level of 5 %.

**Figure 5:**
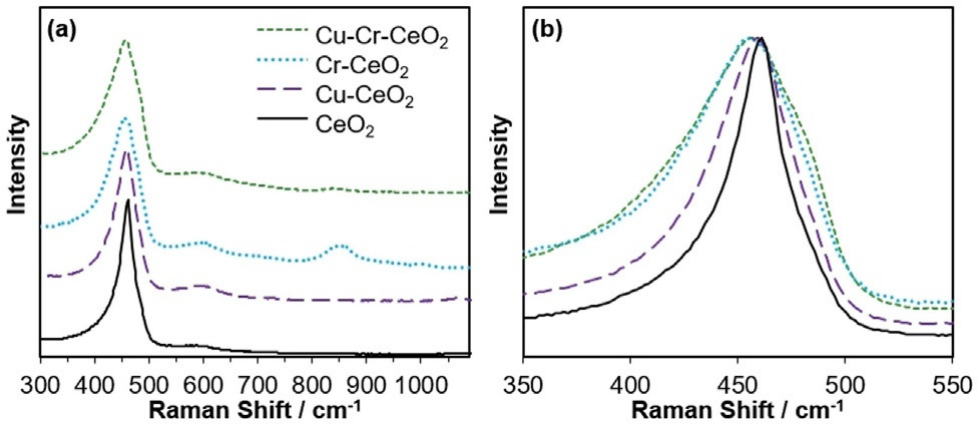
(a) Raman spectra of undoped and doped with 1 % Cu, 1 % Cr, and 1 % Cu/1 % Cr ceria nanorods, and (b) F_2g_ Raman band for the same ceria samples.

Representative Raman spectra of undoped and doped ceria nanorod samples are shown in Figure 5. Ceria has a characteristic Raman band at approximately 465 cm^−1^ (F_2g_ mode), which is attributed to the symmetric vibrational breathing of oxygen surrounding the cerium ions in the fluorite‐type crystal lattice.^[37]^ This band is prominent in all spectra, but for the doped ceria samples it is shifted. There are two main contributions to this shift. First, the substitution of Ce ions with the dopant ions changes the lattice parameters and the oxygen–metal bond length, affecting lattice vibration. Second, differences in oxygen vacancy concentration affect the vibrational mode.[^38^, ^39^] Additionally, a Raman peak broadening is visible in the doped ceria materials, which is associated with changes in crystallite size.^[40]^ This correlates well with the coherent diffraction domain sizes calculated from the XRD spectra in Figure 2, which showed a domain size decrease upon doping. An additional band at approximately 600 cm^−1^ is visible in the doped ceria samples, more prominently than in the case of undoped ceria. This band is generally attributed either to the quantity of oxygen vacancies in the sample or point defects related to the presence of Ce^3+^ ions.^[37]^ Therefore, it can be concluded that the use of copper or chromium dopants increases the quantity of oxygen vacancies in the ceria nanorod samples.

In addition to the characteristic ceria Raman bands, chromium‐doped ceria samples had a prominent band at 860 cm^−1^ and a weak one at 1010 cm^−1^. Both of these are indicative of the presence of Cr^VI^ oxide species interacting with the CeO_2_ surface, instead of being incorporated into ceria's lattice structure.^[41]^ This contrasts with the powder XRD spectrum of Cr‐ceria (Figure 2), in which only ceria diffraction peaks are visible; however, very small (<2 nm) Cr particles may be present that cannot diffract X‐rays. However, EDS analysis (Figures S1–S4) shows that Cr is still highly dispersed across the catalyst.

**Figure 6:**
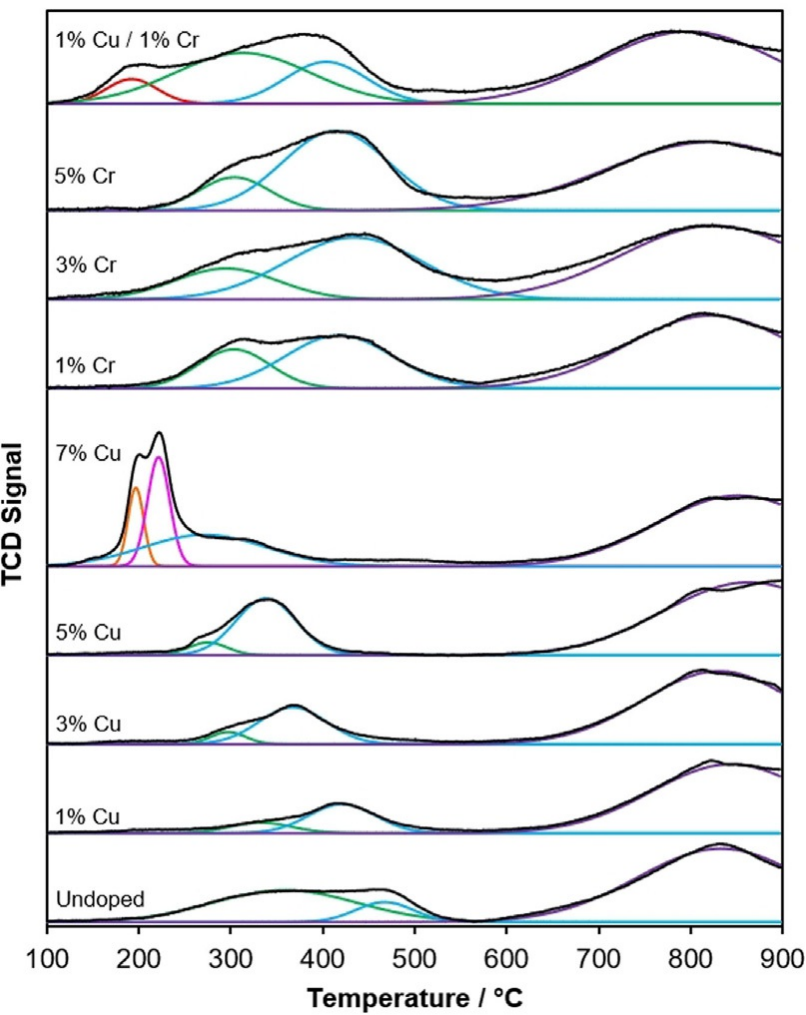
TPR analysis of undoped, Cu, Cr, and Cu/Cr‐doped ceria nanorods.

To understand how Cu and Cr dopants affect the reducibility of the ceria nanorod catalysts, temperature‐programmed reduction (TPR) experiments were performed on the undoped and doped materials (Figure 6). For nanosized ceria materials, two areas of reduction are typically seen: a low‐temperature area (200 to 500 °C) representing consumption of hydrogen due to readily available surface oxygen, and a higher‐temperature area (>600 °C) representing reduction of the bulk lattice oxygen.[^36^, ^42^] In most cases, the deconvolution of the lower temperature reduction area (between 200 and 500 °C) can be represented with two Gaussian peaks (percentage peak fitting error inferior to 3 %). For Cu‐ceria nanorods, the position of these two low‐temperature peaks shifts to the left, from 359 and 467 °C corresponding to undoped ceria nanorods to 275 and 338 °C for the 5 % Cu‐ceria, indicating an increase in surface reducibility at lower temperatures with increasing levels of copper dopant (Table S1). For 1 % Cr‐ceria nanorods, there is also some shift to the left of both peaks, to 303 and 418 °C, but increasing the quantity of Cr dopant does not continue to improve surface reducibility at lower temperatures.

The ratio of low‐temperature to high‐temperature areas in Figure 6, representing the total consumption of hydrogen in surface and bulk reduction, respectively, can be used as a qualitative assessment of the reducibility of the bulk lattice structure, relative to the surface by showing the ratio of readily reducible surface to bulk oxygen.[^30^, ^43^] Doping ceria with other metals generally changes the bulk properties, not just those of the surface.^[17]^ While reactions such as CO oxidation and NO reduction occur on the surface of ceria catalysts, improvements in bulk properties can also improve catalyst performance due to the mobility of oxygen vacancies and vacancy hopping mechanisms.[^44^, ^45^] For undoped ceria the ratio of low‐temperature to high‐temperature areas is 0.47 (Table 1). With increasing levels of copper dopant, this ratio shifts in favor of the high‐temperature area (with a ratio of 0.24 to 0.34), indicating that relative to the surface, the bulk structure becomes more reducible. However, for chromium‐doped ceria, the opposite trend is seen: the bulk becomes *less* reducible relative to the surface, with a ratio of 0.70 to 0.86. This is potentially due to improvement of the surface reducibility and not the bulk or an overall decrease in bulk reducibility.

**Table 1:** Surface concentration of Ce^3+^ in ceria nanorods, calculated from the Ce 3d XPS spectra, and relative peak areas of O_α_, O_β_ and O_γ_ in O 1s XPS spectra. H_2_ TPR result.

Material	Ce^3+^ [at. %]	O_α_ [at. %]	O_β_ [at. %]	O_γ_ [at. %]	Low T/High T TPR peak area ratio
Undoped CeO_2_	22	50.8	28.9	20.2	0.47
1 wt % Cu	23	37.9	40.9	21.3	0.24
3 wt % Cu	23	41.4	36.0	22.6	0.27
5 wt % Cu	26	39.8	38.5	21.7	0.31
7 wt % Cu	27	32.5	38.3	29.2	0.34
1 wt % Cr	16	34.2	50.1	15.8	0.70
3 wt % Cr	15	35.5	49.0	15.5	0.86
5 wt % Cr	16	40.5	42.8	16.7	0.76
1 wt % each Cu/Cr	33	42.5	34.4	23.1	1.20

Two additional sharp and intense reduction peaks appear in the 7 % Cu‐ceria nanorods, centered at 197 and 222 °C. These peaks can be attributed to the reduction of copper particles, segregated from the ceria phase due to the high dopant content.[^46^, ^47^, ^48^] Because separate copper oxide diffraction peaks were not seen until dopant levels reached 9 % (Figure 2), these particles observed at 7 % Cu must be too small and dispersed to significantly diffract X‐rays.

The TPR reduction profile of the simultaneous Cu‐ and Cr‐doped ceria nanorods differs from the singly doped materials. Although there are still distinct low‐temperature and high‐temperature areas of reduction, the low‐temperature reduction area is wider and extends further to lower temperature values in the case of the Cu−Cr doped ceria. Unlike the singly doped nanorod samples, to keep the fitting error inferior to 3 %, the deconvolution of this region is represented by three Gaussian peaks instead of two. The lowest‐temperature peak is centered at 192 °C. This is a strong indication that the two‐dopant material has much‐improved surface reducibility relative to the single copper‐doped ceria nanorods—a synergistic improvement that cannot be explained by the cumulative or average effect of single‐dopant contributions.

Additionally, the ratio of the surface to bulk oxygen reduction areas is 1.2, much higher than that of undoped or singly doped ceria materials. This could also indicate an improvement in surface reducibility, or instead, similarly to Cr‐doped ceria materials, the two‐dopant system might not have improved the oxygen bulk reducibility but only that of the surface.

**Figure 7:**
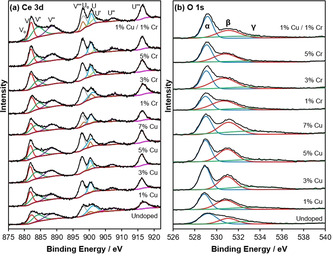
(a) Ce 3d and (b) O1s XPS spectra for Cu, Cr, and Cu/Cr‐doped ceria nanorods.

**Figure 8:**
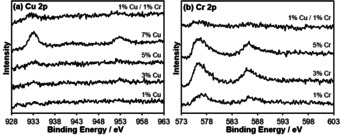
(a) Cu 2p and (b) Cr 2p XPS spectra for Cu, Cr, and Cu/Cr‐doped ceria nanorods.

X‐ray photoelectron spectroscopy (XPS) analysis was used to gain further insight into the surface state of the ceria nanorods. XPS spectra of Ce 3d, O 1s, Cu 2p, and Cr 2p are shown in Figures 7 and 8. In Figure 7 a, the Ce 3d XPS spectra consist of five peaks (Uo, U, U′, U′′, and U′′′) representing 3d_3/2_ and five peaks (Vo, V, V′, V′′, and V′′′) representing 3d_5/2_. According to previously published methods, Vo, V′, Uo, and U′ can be assigned to Ce^3+^, while the remaining peaks (V, V′′, V′′′, U, U′′, and U′′′) can be assigned to Ce^4+^, and the relative ratio of peak areas can be used to determine the percentage concentration of Ce^3+^ and Ce^4+^ at the surface.[^30^, ^37^, ^42^] This is shown in Table 1. This analysis reveals that undoped ceria nanorods have a nonstoichiometric surface, with 22 % of Ce 3d photoemission due to Ce^3+^ and 78 % due to Ce^4+^. The addition of low levels of Cu dopant (1 and 3 %) only slightly increases the concentration of Ce^3+^, but higher levels of Cu (5 and 7 %) result in a higher level of surface reduction, with the percentage of Ce^3+^ increasing to 26 and 27 %, respectively. In contrast, the addition of Cr dopant decreases the percentage of Ce^3+^ at the surface. Both results corroborate the TPR analysis above; in summary, Cu improves ceria's surface reducibility, whereas Cr does not have a relevant effect. Cu/Cr‐doped ceria nanorods demonstrate the highest level of surface reduction, with the concentration of Ce^3+^ calculated to be 33 %. This phenomenon of enhanced surface reducibility with low levels of both Cu and Cr dopant also supports the TPR analysis in Figure 6.

XPS spectra in the O 1s region are shown in Figure 7 b for the undoped and doped ceria nanorods, while relative peak areas are reported in Table 1. The O 1s spectrum is broad and consists of peak contributions from the various oxygen species at the surface of ceria. These spectra were resolved with three Gaussian‐Lorentzian peaks. The peak at approximately 529 eV (O_α_) can be attributed to the lattice oxygen of Ce^4+^, while the peak at 531 eV (O_β_) can be attributed to oxygen vacancies or the lower‐coordination lattice oxygen of Ce^3+^. This is significant because reduced Ce^3+^ defects and oxygen vacancies are understood to be the active sites on ceria‐catalyzed surface reactions.^[49]^ CO oxidation on ceria surfaces proceeds via the Mars van Krevelen mechanism: CO molecules adsorb onto the ceria surface and react with a surface oxygen atom to form an intermediate. This intermediate desorbs as CO_2_, leaving behind an oxygen vacancy, which is compensated by the reduction of Ce^4+^ to Ce^3+^.[^50^, ^51^, ^52^]

However, O_β_ can also be associated with surface adsorbed oxygen and hydroxy groups. The broad peak at 533 eV (O_γ_) is also associated with these surface oxygen species.[^30^, ^37^] Prior work on similarly prepared ceria nanorods demonstrated (via DRIFTS experiments) that hydroxy and carbonate species were present on the surface of the ceria nanorods and that the presence of hydroxy‐containing groups was correlated with catalytic activity for oxidation reactions of naphthalene and toluene.^[30]^ it was tentatively proposed that these OH sites act as adsorption points in the first reaction steps at low temperatures.

No shift in the O 1s binding energy is evident when comparing the undoped and doped ceria samples. However, the undoped ceria spectrum has a larger contribution from stoichiometric lattice oxygen (O_α_) than the doped materials: approximately 50 % of the total area of the three peaks compared with, for example, 38, 34, and 43 % for 1 % Cu, 1 % Cr, and 1 % Cu/1 % Cr‐ceria, respectively.

**Figure 9:**
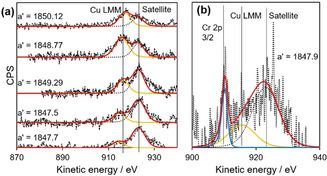
Cu LMM spectra for (a) Cu‐doped ceria nanorods (from top to bottom; 1, 3, 5, 7, 9 % Cu‐ceria) and (b) 1 % Cu/1 % Cr ceria nanorods.

As shown in Figure 8 a, no Cu 2p peaks are observable in the 1 % Cu‐doped ceria sample by XPS. Weak Cu 2p_1/2_ and Cu 2p_3/2_ peaks are visible in the 3 % and 5 % Cu‐ceria, while the 7 % Cu‐ ceria shows strong Cu 2p_1/2_ and Cu 2p_3/2_ peaks at 933.0 and 952.8 eV, respectively. In contrast to 1 % Cu‐ceria, the 1 % Cu/1 % Cr‐ceria has observable Cu 2p^1/2^ and Cu 2p^3/2^ peaks, indicating that the co‐doped ceria has a higher concentration of Cu at the surface than the singly doped 1 % Cu‐ceria. Analysis of both the Cu 2p and Cu LMM region (Figure 9) was employed to reliably determine copper speciation, using the modified auger parameter (α′). The Cu LMM feature was deconvoluted and kinetic energy determined and α′ was found to range between 1850.12 and 1847.7 eV, consistent with ionic copper species.^[53]^ Furthermore, there appeared to be an inversely proportional relationship between α′ and copper loading, with lower loadings reporting a lower modified auger parameter. This lowering of α′ can be attributed to decreased average screening due to a decrease in copper particle size and the interaction between the copper and the highly polarizable support.^[54]^ Due to the lack of a clear shakeup satellite peak in the Cu 2p spectra at 942 eV, characteristic of Cu^2+^, the samples exposure to air, and the Cu_2_O XRD peaks observed for 9 % Cu‐ceria, Cu 2p XPS peaks can be assigned to Cu^+^. Figure 8 b shows the Cr 2p XPS spectra for Cr and Cu/Cr‐doped ceria nanorods. The visible Cr 2p peaks at 576 and 586 eV are assigned to Cr^3+^ and the shoulder peaks at 580 and 589 eV to Cr^6+^.^[55]^ Intensity is higher for the 3 % and 5 % Cr‐ceria. Unlike Cu, the XPS spectrum for 1 % Cu/1 % Cr‐ceria shows a weaker Cr intensity than 1 % Cr‐ceria.

**Figure 10:**
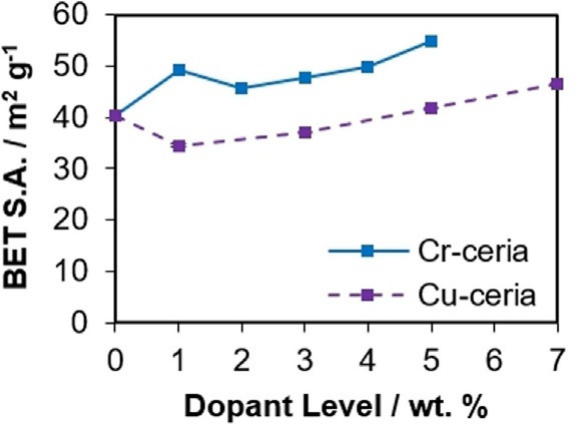
BET surface area vs. dopant level for Cu and Cr doped ceria.

Nitrogen adsorption experiments show that the ceria nanorods, both undoped and doped, are non‐porous but mesoporosity is observed due to interparticle voids, with a type IV isotherm in all cases (Figure S6).^[56]^ BET surface area, measured at low relative pressures, is in the range 34 to 55 m^2^ g^−1^ (Figure 10). The presence of Cu has a negligible effect on the surface area, with no clear overall trend. For Cr‐doped ceria, all doped samples had higher surface areas than undoped ceria nanorods.

**Figure 11:**
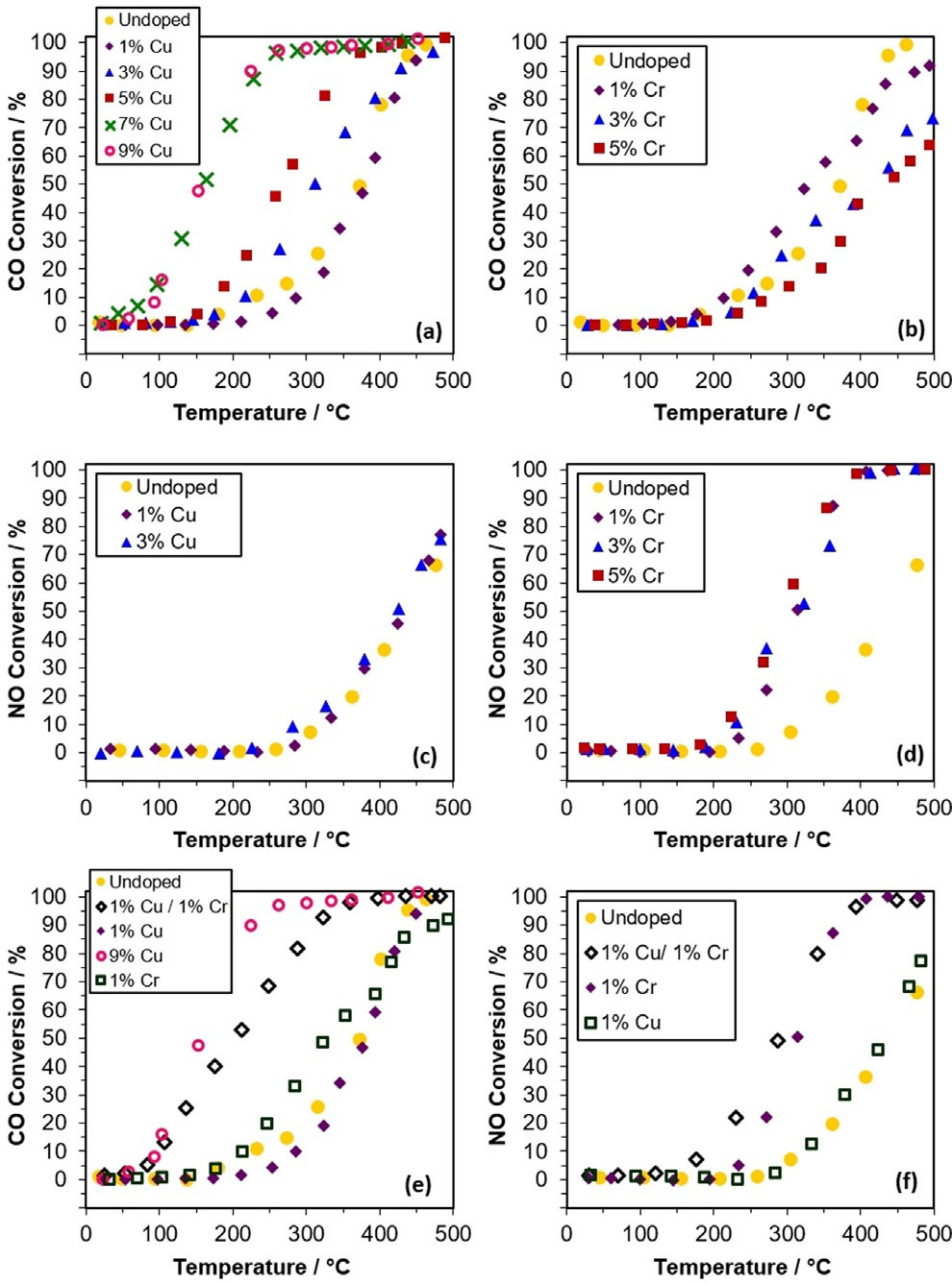
CO oxidation catalytic conversions for (a) Cu‐doped and (b) Cr‐doped ceria nanorods. NO reduction catalytic conversions for (c) Cu‐doped and (d) Cr‐doped ceria nanorods. Conversion of two‐dopant Cu/Cr doped ceria nanorods for (e) CO oxidation and (f) NO reduction.

CO oxidation and NO reduction on the doped ceria nanorods were tested over cycles of six steps between room temperature and 500 °C and representative stability data is shown in Figure S7 and S8. The CO oxidation at various temperatures was stable. The NO reduction was stable for the first three steps, and then slightly decreased for the following steps, but samples were still active. Figure 11 shows the averaged catalytic conversion for the first three steps. Copper doping of ceria significantly improves the low‐temperature catalytic CO oxidation with respect to undoped ceria rods, with conversion increasing as the Cu loading increases up to 7 % Cu above which no further improvements are observed (Figure 11 a). Similar trends in improved CO conversion on ceria catalysts have been previously observed with transition metal doping.[^57^, ^58^, ^59^] The large jump in CO conversion between the 5 % Cu and 7 % Cu ceria catalysts can be explained with the appearance of the separate copper oxide phase at 7 % Cu loading (and at 9 % in powder XRD), seen in the TPR analysis. Ceria‐supported copper is known to be an effective low‐temperature oxidative catalyst.[^60^, ^61^] However, copper and other base metals are not used in automotive catalysis due to sulfur poisoning and thermal durability issues, so despite the enhanced CO conversion, a separate copper phase is not desirable.^[3]^ A further increase in copper loading from 7 to 9 % did not result in an improvement in low‐temperature CO conversion. This could be attributed to the doping limit being reached somewhere between 5 and 7 % Cu, and the additional copper oxide particles in the 9 % sample (visible in XRD) not providing more catalytic CO conversion than the copper oxide particles present in the 7 % Cu‐ceria nanorod material (as confirmed by TPR). In contrast to copper, doping ceria nanorods with 1 % Cr slightly increases its catalytic CO oxidation at low temperatures but decreases it at all temperatures for higher Cr percentages, compared with undoped ceria nanorods (Figure 11 b). This also correlates with TPR and XPS results, which suggest that chromium doping of ceria mostly decreased the reducibility of the material and the concentration of Ce^3+^ at the surface.

Regarding NO reduction (Figure 11 c,d), Cr‐doped ceria nanorods are effective at improving conversions beyond that of pure ceria, while Cu‐doped ceria do not show improvement compared with undoped ceria nanorods. This is expected in view of the characterization: Cu doping improves ceria's oxidative capability by increasing the reducibility of ceria, but this can also make the healing of oxygen vacancies somewhat less favorable.^[29]^ In contrast, chromium demonstrates the opposite effect. However, chromium doping at levels above 1 % do not show further improvement in NO reduction conversions. As with CO oxidation, this activity matches the analysis of the Ce 3d XPS spectra—ceria nanorods doped with 1, 3, and 5 % Cr show very similar levels of surface reduction.

Catalyst performance of the 1 % Cu/1 % Cr‐ceria nanorod material is shown in Figure 11 e for CO oxidation and Figure 11 f for NO reduction. Despite containing only 1 % each Cu and Cr, the two‐dopant ceria nanorods show CO oxidation that is similar to the 7 and 9 % Cu ceria nanorods, as well as slightly improved NO reduction compared with 1 % Cr‐ceria. Therefore, co‐doped Cu−Cr ceria appears to provide a unique synergistic effect, with a CO oxidation close to the one of 9 % Cu‐ceria nanorods, despite only containing 1 % Cu (2 % total dopant content). At the same time, the co‐dopant catalyst retains NO reduction conversions similar to that of the Cr‐ceria nanorods, with a slightly improved T_10_ value. “Synergistic” in this case can be defined as showing improvement in reactant conversions beyond what an equivalent level of single‐metal dopant could be responsible for.

The ceria nanorods of this work preferentially expose (110) ceria facets.^[30]^ Assuming Cu and Cr atoms are incorporated similarly on (110) and (111) ceria surfaces, DFT simulations by Yoshida et al. can shed some light on such synergy in 1 % Cu/1 % Cr‐ceria nanorod.[^64^, ^66^] They report that when Ce on the (111) surface of ceria is substituted with Cu, the dominant species is Cu^2+^. However, the substitution of Ce with Cu and Cr results in the dominant Cu species being Cu^+^. The presence of Cu^+^ in doped ceria is known to enhance CO chemisorption on the catalyst surface and catalytic activity for CO oxidation.^[67]^ In this case, CO adsorbs on the surface at Cu^+^ sites and react with neighboring oxygen to form CO_2_ and create oxygen vacancies, which are balanced by the reduction of Ce^4+^ to Ce^3+^. Conversely, for the reduction of NO, the presence of Cr enhances electron density on neighboring Ce sites and lowers the barrier for oxygen vacancy formation, both of which improve NO adsorption on Ce sites near Cr on the doped ceria surface.[^68^, ^69^] This corroborates the enhanced reducibility of the Cu/Cr‐ceria seen in TPR and XPS analysis as well as the significantly improved catalytic performance for CO oxidation compared with singly doped ceria.

For NO reduction, the 1 % Cu/1 % Cr‐ceria catalyst showed improvement compared with 1 % Cr‐ceria, even though the addition of copper alone did not improve conversions over undoped ceria. This can be attributed to the combination of the influence of both dopants on the properties of the catalyst. A higher concentration of oxygen vacancies is known to enhance the reduction of NO to N_2_ by promoting the disassociation of NO.[^70^, ^71^] However, improved NO adsorption is also necessary to improve overall catalytic activity. In the 1 % Cu/1 % Cr‐ceria catalyst, Cu provides additional oxygen vacancies while Cr enhances NO adsorption on Ce^3+^ sites on the catalyst surface, and in combination this provides additional catalytic conversions beyond that of either singly doped catalyst. Conversely, for the singly doped Cu‐ceria catalysts, while Cu will still enhance oxygen vacancy concentration and surface reducibility, NO adsorption on the catalyst surface is not necessarily improved, so no overall improvement in catalytic conversion is seen.

Therefore, while doping ceria with copper improves CO oxidation conversions but inhibits NO reduction and doping ceria with chromium has the opposite effect, the simultaneous co‐doping of ceria nanorods with both Cu and Cr retains the benefits of each single dopant while significantly enhancing CO oxidation, a phenomenon that has not yet been reported in the literature for this doped system. These results also show that the improved reducibility provided by copper doping does not necessarily have a negative effect on the healing of oxygen vacancies. Co‐doping is necessary for this synergistic effect—a physical mixture of Cu‐ceria and Cr‐ceria shows much lower catalytic CO oxidation (Figure S9).

Related studies of NO reduction with CO on supported metals on ceria (MO_x_/CeO_2_) prepared via incipient wetness impregnation have shown the importance of controlling the loading amount to maximize the number of metal–oxygen–support interfacial bonds.[^72^, ^73^, ^74^, ^75^, ^76^, ^77^] Maximum conversions and activities are typically found at approximately the monolayer coverage, avoiding the presence of separate crystallites phases that would be detectable by for example XRD. Such results are similar to these presented here, in which small and controlled amounts of dopants lead to optimal results, especially in 1 % Cu/1 % Cr‐ceria that has a higher concentration of Cu at the surface than the singly doped 1 % Cu‐ceria according to XPS.

Various other ceria catalysts doped with copper or chromium have recently been reported.[^14^, ^21^, ^22^, ^61^, ^62^] Direct comparisons with other articles is difficult due to differences in reaction conditions such as catalyst loading and reactant flowrates or ratios, which widely vary between different research groups. Nevertheless, conversions can be normalized in terms of rate, shown in Table 2 for CO oxidation and NO reduction along with *T*_10_ and *T*_50_ values (the temperature at which 10 % and 50 % conversion of CO or NO is achieved). While the rates of our catalyst is similar to or better than many other reported doped ceria materials, even those with higher dopant content, the low‐temperature activity for CO oxidation is not as good as that of the 3.9 % Cu‐doped ceria nanorods reported in Table 2.^[21]^ This can be attributed to the higher copper loading and improvements in BET surface area (72 m^2^ g^−1^), and porosity due to their MOF‐templated synthesis, compared with our hydrothermally prepared Cu/Cr‐doped nanorods. In contrast, for NO reduction, our Cu/Cr‐doped ceria nanorods performed slightly better at 150 °C but somewhat worse at 200 °C compared with a Cr‐doped ceria catalyst (Ce_20_Cr_1_O_x_) or a 0.07 % each Cu/Cr deposited on ceria (Table 2).^[22]^ In any case, the latter uses a pulsed cathodic arc plasma technique in vacuum, much more complicated than our hydrothermal approach.

**Table 2:** Comparison of copper‐ceria and chromium‐ceria catalysts for CO oxidation and NO reduction.

Catalyst	Reaction	*T*_10_ [°C]	*T*_50_ [°C]	Rate [μmol g^−1^ min^−1^]		Ref.
				At 50 °C	At 100 °C	
1 % each Cu/Cr co‐doped ceria nanorods	CO ox.	100	206	8.2	27.3	this work
3.9 % Cu‐doped ceria nanorods	CO ox.	65	122	61.3	352.7	[21]
3.9 % Cu‐doped ceria nanospheres	CO ox.	142	232	0.0	13.1	[61]
2.0 % Cu‐doped ceria nanopolyhedra	CO ox.	113	161	0.0	1.2	[14]
8.5 % Cu deposited on ceria nanorods	CO ox.	40	73	16.4	90.0	[62]
0.07 % each Cu/Cr deposited on ceria	CO ox.	86	120	0.0	18.0	[63]

Catalyst	Reaction	*T*_10_ [°C]	*T*_50_ [°C]	Rate [μmol g^−1^ min^−1^]		Ref.
				At 150 °C	At 200 °C	
1 % each Cu/Cr co‐doped ceria nanorods	NO red.	194	243	6.1	26.6	this work
Ce_20_Cr_1_O_x_	NO red.	202	216	0.0	40.9	[22]
0.07 % each Cu/Cr deposited on ceria	NO red.	143	300	17.2	45.4	[64]

## Conclusions

Copper and chromium have been used as dopants for ceria nanorod catalysts produced via a hydrothermal synthesis method. Doping does not significantly affect the nanorods shape. Metal particles different to ceria and large enough to diffract X‐rays in our conditions are only observed for copper levels above 7 %. The experimental characterization confirms the incorporation of Cu or Cr in the ceria crystal lattice, increase of surface oxygen vacancies, and changes in the surface and bulk reducibility. When tested as catalysts, copper doping significantly improves the CO oxidation conversions but not for NO reduction, while the opposite is true for chromium‐doped ceria. However, simultaneously doping a ceria nanorod catalyst with both Cu and Cr dopants results in a catalyst which shows improved conversions for both CO oxidation and NO reduction compared with undoped ceria nanorods. Additionally, the use of co‐dopants appears to provide a synergistic effect, with only 1 % Cu and 1 % Cr required to provide CO oxidation conversions like 7 % Cu‐doped ceria nanorods. This behavior is supported by TPR and XPS analyses which show improved low‐temperature surface reducibility in co‐doped Cu/Cr‐ceria compared with either Cu or Cr singly doped ceria materials. Cu XPS moreover shows a higher surface Cu concentration, which based on DFT calculations in literature are expected to be mainly Cu^+^, known to enhance CO chemisorption on the catalyst surface and therefore catalytic CO oxidation. In a nutshell, Cu/Cr‐doped ceria nanorods show promise as an improved catalyst for both oxidative and reductive reactions. This research demonstrates the potential effectiveness of co‐doping with transition metals as a strategy for improving the effectiveness of ceria‐based catalysts. Future research should be focused on the optimization of dopant levels, examination of other dopant metals, and testing these catalysts under more typical real‐world conditions such as after oxidative pre‐treatments. It would also be useful to research the N_2_ selectivity for the NO reduction with CO in these doped materials.

## Experimental Section

A facile hydrothermal method was used to synthesize ceria nanorods, both undoped and doped.^[36]^ 120 mL of a 15 m aqueous sodium hydroxide solution and 1.8 g of Ce(NO_3_)_3_⋅6 H_2_O were added to a 150 mL Teflon‐lined stainless‐steel autoclave. For doped ceria, a necessary amount of the respective dopant precursor in aqueous solution—0.25 m CuSO_4_ or 0.3 m Cr(NO_3_)_3_—was added to the Teflon‐lined autoclave at this time to achieve the desired wt % dopant (for example, 0.448 mL of CuSO_4_ solution was added to achieve 1 % Cu‐ceria). The mixture was stirred vigorously for 20 s. The autoclave was sealed and heated at 100 °C in an air‐circulating oven at a rate of 5 °C min^−1^, held at this temperature for 10 h, after which it was allowed to cool naturally to room temperature. Post‐synthesis, the product was separated from the sodium hydroxide solution via vacuum filtration and washed with approximately 500 mL of deionized water. The wet product was dried in a vacuum oven at 80 °C overnight. The resulting dry powder was gently grounded in an agate mortar and pestle. Highly crystalline ceria nanorods, free of organic matter, were obtained following this approach. No calcination was, therefore, carried out. Samples were labelled according to the wt % of metal dopant used in the synthesis.

XRD was performed with a Bruker D8‐Advance using a Cu_Kα_ radiation source. Measurements were taken from 20 to 90° 2*θ* with a step size of 0.0164° 2*θ* and 0.25 sec per step. Coherent diffraction domain sizes were calculated with the Scherrer equation. Catalyst surface areas were calculated with nitrogen adsorption experiments using a Micromeritics 3Flex Surface Characterization Analyzer. Samples were degassed under vacuum for 90 minutes at 200 °C. The surface areas were calculated using the BET. XPS analysis was performed using a Kratos SUPRA XPS instrument with monochromated Al_Kα_ X‐rays (1486.69 eV). Survey scans were recorded with a pass energy of 160 eV, while high resolution spectra were recorded with a pass energy of 20 eV. TEM was performed using a JEOL JEM2100Plus microscope. Raman microscopy was performed using a Renishaw inVia Raman Microscope, using a 532 nm green laser with 10 s exposure time and 1 % laser power. TPR experiments were performed with a Micromeritics AutoChem II 2920 equipped with a thermal conductivity detector; 0.1 g of each sample was degassed under helium and consequently heated from room temperature to 900 °C at a rate of 10 °C min^−1^, under a flow of 50 mL min^−1^ of 5 % hydrogen in argon.

The catalytic CO oxidation and NO reduction were performed with a U‐shaped quartz tube reactor (10 mm ID). In a typical experiment, a catalytic bed of 4 cm^3^ silicon carbide particles was used, with 15 mg ceria nanorod catalyst dispersed throughout. Within the quartz tube reactor, the catalytic bed was secured at both ends with high‐temperature quartz wool. For CO oxidation, the reactant feed consisted of 2000 ppm CO and 2000 ppm O_2_ in nitrogen. The total flow rate was 50 mL min^−1^, achieving a weight hourly space velocity (WHSV) of 200 L g^−1^ h^−1^. For NO reduction using CO as the reductant, the reactant feed consisted of 1667 ppm NO and 2667 ppm CO in nitrogen. The total flow rate was 45 mL min^−1^, achieving a WHSV of 180 L g^−1^ h^−1^. For both CO oxidation and NO reduction, the catalysts were tested though cycles of six steps: (step 1) the temperature was increased from 20 to 500 °C with a ramp rate of 2.5 °C min^−1^, (step 2) kept at 500 °C for 30 min, (step 3) cooled down with a ramp rate again of 2.5 °C min^−1^, and then (step 4–6) heated and cooled again in the same manner (total time: 830 min). No pretreatment of the catalysts was carried out. The outlet gas during the whole process was analyzed with a Hiden mass spectrometer and a Fuji Electric ZRH Infrared Gas Analyzer (for CO). Note both N_2_O and CO_2_ have the same *m*/*z* value, so we could not distinguish them for selectivity studies.

Experimental data are available via the University of Bath Research Data Archive (DOI: https://doi.org/10.15125/BATH‐00605).

## Conflict of interest

The authors declare no conflict of interest.

## Supporting information

As a service to our authors and readers, this journal provides supporting information supplied by the authors. Such materials are peer reviewed and may be re‐organized for online delivery, but are not copy‐edited or typeset. Technical support issues arising from supporting information (other than missing files) should be addressed to the authors.

Supplementary

## References

[1] M. Melchionna , P. Fornasiero , Mater. Today 2014, 17, 349–357.

[2] T. Montini , M. Melchionna , M. Monai , P. Fornasiero , Chem. Rev. 2016, 116, 5987–6041. 27120134 10.1021/acs.chemrev.5b00603

[3] M. V. Twigg , Catal. Today 2011, 163, 33–41.

[4] C. H. Bartholomew , R. J. Farrauto , Fundamentals of Industrial Catalytic Processes, Wiley-Blackwell, 2006.

[5] M. S. Reiter , K. M. Kockelman , Transport Res. D 2016, 43, 123–132.

[6] M. V. Faria , R. A. Varella , G. O. Duarte , T. L. Farias , P. C. Baptista , Sci. Total Environ. 2018, 630, 544–559. 29494966 10.1016/j.scitotenv.2018.02.232

[7] M. Shelef , R. W. McCabe , Catal. Today 2000, 62, 35–50.

[8] C. L. S. Wiseman , F. Zereini , Sci. Total Environ. 2009, 407, 2493–2500. 19181366 10.1016/j.scitotenv.2008.12.057

[9] A. Gupta , U. V. Waghmare , M. S. Hegde , Chem. Mater. 2010, 22, 5184–5198.

[10] M. Piumetti , T. Andana , S. Bensaid , N. Russo , D. Fino , R. Pirone , Nanoscale Res. Lett. 2016, 11, 165. 27009532 10.1186/s11671-016-1375-z PMC4805670

[11] C. D. Curran , L. Lu , C. J. Kiely , S. McIntosh , J. Mater. Chem. A 2018, 6, 244–255.

[12] M. Mogensen , Solid State Ionics 2000, 129, 63–94.

[13] K. Kim , J. W. Han , Catal. Today 2017, 293–294, 82–88.

[14] M. Dosa , M. Piumetti , S. Bensaid , T. Andana , C. Novara , F. Giorgis , D. Fino , N. Russo , Catal. Lett. 2018, 148, 298–311.

[15] M. Dosa , M. Piumetti , S. Bensaid , N. Russo , D. Fino , Catal. Lett. 2019, 149, 107–118.

[16] H. J. Kim , G. Lee , M. G. Jang , K.-J. Noh , J. W. Han , ChemCatChem 2019, 11, 2288–2296.

[17] Y. Guo , H. Wei , G. Zhao , X. Ma , W. Zhu , Y. Yang , Fuel 2017, 206, 318–324.

[18] X. Liu , L. Han , W. Liu , Y. Yang , Eur. J. Inorg. Chem. 2014, 5370–5377.

[19] W.-X. Tang , P.-X. Gao , MRS Commun. 2016, 6, 311–329.

[20] D. Mukherjee , B. M. Reddy , Catal. Today 2018, 309, 227–235.

[21] S. Li , N. Wang , Y. Yue , G. Wang , Z. Zu , Y. Zhang , Chem. Sci. 2015, 6, 2495–2500. 28706658 10.1039/c5sc00129c PMC5489022

[22] C. Deng , M. Li , J. Qian , Q. Hu , M. Huang , Q. Lin , Y. Ruan , L. Dong , B. Li , M. Fan , Chem. Asian J. 2016, 11, 2144–2156. 27435470 10.1002/asia.201600516

[23] X. Yao , C. Tang , Z. Ji , Y. Dai , Y. Cao , F. Gao , L. Dong , Y. Chen , Catal. Sci. Technol. 2013, 3, 688–698.

[24] C.-Q. Zhu , H. Liang , S.-H. Li , Y.-X. Hong , D.-Q. Ye , Chin. J. Inorg. Chem. 2011, 6, 1093–1100.

[25] B. Weidenhof , M. Reiser , K. Stöwe , W. F. Maier , M. Kim , J. Azurdia , E. Gulari , E. Seker , A. Barks , R. M. Laine , J. Am. Chem. Soc. 2009, 131, 9207–9219. 19566095 10.1021/ja809134s

[26] X. Wu , Q. Liang , D. Weng , J. Fan , R. Ran , Catal. Today 2007, 126, 430–435.

[27] S. Datta , L. Torrente-Murciano , Curr. Opin. Chem. Eng. 2018, 20, 99–106.

[28] M. D. Krcha , A. D. Mayernick , M. J. Janik , J. Catal. 2012, 293, 103–115.

[29] M. Nolan , J. Chem. Phys. 2009, 130, 144702. 19368460 10.1063/1.3110702

[30] J. M. López , A. L. Gilbank , T. García , B. Solsona , S. Agouram , L. Torrente-Murciano , Appl. Catal. B 2015, 174, 403–412.

[31] H.-X. Mai , L.-D. Sun , Y.-W. Zhang , R. Si , W. Feng , H.-P. Zhang , H.-C. Liu , C.-H. Yan , J. Phys. Chem. B 2005, 109, 24380–24385. 16375438 10.1021/jp055584b

[32] M. Nolan , S. C. Parker , G. W. Watson , Surf. Sci. 2005, 595, 223–232.

[33] T. Vinodkumar , B. G. Rao , B. M. Reddy , Catal. Today 2015, 253, 57–64.

[34] H. Yu , J. Yu , S. Liu , S. Mann , Chem. Mater. 2007, 19, 4327–4334.

[35] R. D. Shannon , Acta Crystallogr. Sect. A 1976, 32, 751–767.

[36] L. Torrente-Murciano , A. Gilbank , B. Puertolas , T. Garcia , B. Solsona , D. Chadwick , Appl. Catal. B 2013, 132–133, 116–122.

[37] A. Younis , D. Chu , Y. V. Kaneti , S. Li , Nanoscale 2016, 8, 378–387. 26616275 10.1039/c5nr06588g

[38] D. Mukherjee , B. G. Rao , B. M. Reddy , Top. Catal. 2017, 60, 1673–1681.

[39] Y. She , Q. Zheng , L. Li , Y. Zhan , C. Chen , Y. Zheng , X. Lin , Int. J. Hydrogen Energy 2009, 34, 8929–8936.

[40] S. Saitzek , J. F. Blach , S. Villain , J. R. Gavarri , Phys. Status Solidi A 2008, 205, 1534–1539.

[41] F. D. Hardcastle , I. E. Wachs , J. Mol. Catal. 1988, 46, 173–186.

[42] H. Sohn , G. Celik , S. Gunduz , D. Dogu , S. Zhang , J. Shan , F. F. Tao , U. S. Ozkan , Catal. Lett. 2017, 147, 2863–2876.

[43] M. Lykaki , E. Papista , N. Kaklidis , S. A. C. Carabineiro , M. Konsolakis , Catalysts 2019, 9, 233. 10.3390/nano9121739 PMC6955880 31817667

[44] M. Nolan , J. E. Fearon , G. W. Watson , Solid State Ionics 2006, 177, 3069–3074.

[45] Z.-K. Han , Y.-G. Wang , Y. Gao , Chem. Commun. 2017, 53, 9125–9128. 10.1039/c7cc04440b 28759079

[46] D. G. Araiza , A. Gómez-Cortés , G. Díaz , Catal. Today 2017, 282, 185–194.

[47] W.-P. Dow , Y.-P. Wang , T.-J. Huang , Appl. Catal. A 2000, 190, 25–34.

[48] J. Papavasiliou , M. Rawski , J. Vakros , G. Avgouropoulos , ChemCatChem 2018, 10, 2096–2106.

[49] W. C. Chueh , A. H. McDaniel , M. E. Grass , Y. Hao , N. Jabeen , Z. Liu , S. M. Haile , K. F. McCarty , H. Bluhm , F. El Gabaly , Chem. Mater. 2012, 24, 1876–1882.

[50] K. Wu , L.-D. Sun , C.-H. Yan , Adv. Energy Mater. 2016, 6, 1600501.

[51] V. Shapovalov , H. Metiu , J. Catal. 2007, 245, 205–214.

[52] B. Liu , W. Li , W. Song , J. Liu , Phys. Chem. Chem. Phys. 2018, 20, 16045–16059. 29850757 10.1039/c8cp01694a

[53] M. C. Biesinger , Surf. Interface Anal. 2017, 49, 1325–1334.

[54] J. Batista , A. Pintar , J. P. Gomilšek , A. Kodre , F. Bornette , Appl. Catal. A 2001, 217, 55–68.

[55] Y. Chen , D. An , S. Sun , J. Gao , L. Qian , Materials 2018, 11, 269. 10.3390/ma11020269 PMC5848966 29425145

[56] K. S. W. Sing , D. H. Everett , R. A. W. Haul , L. Moscou , R. A. Pierotti , J. Rouquérol , T. Siemieniewska , Pure Appl. Chem. 1982, 54, 2201–2218.

[57] L. Zhou , X. Li , Z. Yao , Z. Chen , M. Hong , R. Zhu , Y. Liang , J. Zhao , Sci. Rep. 2016, 6, 23900. 27030159 10.1038/srep23900 PMC4814921

[58] J. S. Elias , K. A. Stoerzinger , W. T. Hong , M. Risch , L. Giordano , A. N. Mansour , Y. Shao-Horn , ACS Catal. 2017, 7, 6843–6857.

[59] Y. Park , S. K. Kim , D. Pradhan , Y. Sohn , Chem. Eng. J. 2014, 250, 25–34.

[60] M. Konsolakis , Appl. Catal. B 2016, 198, 49–66.

[61] K. Kappis , C. Papadopoulos , J. Papavasiliou , J. Vakros , Y. Georgiou , Y. Deligiannakis , G. Avgouropoulos , Catalysts 2019, 9, 138.

[62] F. Yang , J. Wei , W. Liu , J. Guo , Y. Yang , J. Mater. Chem. A 2014, 2, 5662–5667.

[63] M. Lykaki , E. Pachatouridou , S. A. C. Carabineiro , E. Iliopoulou , C. Andriopoulou , N. Kallithrakas-Kontos , S. Boghosian , M. Konsolakis , Appl. Catal. B 2018, 230, 18–28.

[64] H. Yoshida , N. Yamashita , S. Ijichi , Y. Okabe , S. Misumi , S. Hinokuma , M. Machida , ACS Catal. 2015, 5, 6738–6747.

[65] H. Yoshida , Y. Okabe , N. Yamashita , S. Hinokuma , M. Machida , Catal. Today 2017, 281, 590–595.

[66] H. Yoshida , Y. Okabe , S. Misumi , H. Oyama , K. Tokusada , S. Hinokuma , M. Machida , J. Phys. Chem. C 2016, 120, 26852–26863.

[67] Z.-Y. Pu , X.-S. Liu , A.-P. Jia , Y.-L. Xie , J.-Q. Lu , M.-F. Luo , J. Phys. Chem. C 2008, 112, 15045–15051.

[68] K. Koizumi , H. Yoshida , M. Boero , K. Tamai , S. Hosokawa , T. Tanaka , K. Nobusada , M. Machida , Phys. Chem. Chem. Phys. 2018, 20, 25592–25601. 30131992 10.1039/c8cp04314k

[69] K. Koizumi , K. Nobusada , M. Boero , Phys. Chem. Chem. Phys. 2017, 19, 3498–3505. 27901152 10.1039/c6cp05957k

[70] X. Niu , Z. Lei , C. Yang , New J. Chem. 2019, 43, 18611–18618.

[71] S. Roy , M. S. Hegde , G. Madras , Appl. Energy 2009, 86, 2283–2297.

[72] Y. Wang , A. Zhu , Y. Zhang , C. T. Au , X. Yang , C. Shi , Appl. Catal. B 2008, 81, 141–149.

[73] D. Prieto-Centurion , T. R. Eaton , C. A. Roberts , P. T. Fanson , J. M. Notestein , Appl. Catal. B 2015, 168–169, 68–76.

[74] T. C. Peck , G. K. Reddy , M. Jones , C. A. Roberts , J. Phys. Chem. C 2017, 121, 8435–8443.

[75] S. Zhang , Y. Li , J. Huang , J. Lee , D. H. Kim , A. I. Frenkel , T. Kim , J. Phys. Chem. C 2019, 123, 7166–7177.

[76] S. Zhang , T. Kim , Mol. Catal. 2020, 494, 111123.

[77] L. Savereide , S. L. Nauert , C. A. Roberts , J. M. Notestein , J. Catal. 2018, 366, 150–158.

