# Supplementary material for: Synergistic Effect of Simultaneous Doping of Ceria Nanorods with Cu and Cr on CO Oxidation and NO Reduction
Source: Chemistry. 2020 Dec 27;27(6):2165–74. doi: 10.1002/chem.202004623 (PMC7898804; doi:10.1002/chem.202004623)
Supplement: Supplementary file 1 — Supplementary [file CHEM-27-2165-s001.pdf]

Chemistry–A European Journal

Supporting Information

Synergistic Effect of Simultaneous Doping of Ceria Nanorods with Cu and Cr on CO Oxidation and NO Reduction

Shawn C. Rood,[a] Oriol Pastor-Algaba,[b] Albert Tosca-Princep,[b] Bruno Pinho,[c] Mark Isaacs,[d] Laura Torrente-Murciano,*[c] and Salvador Eslava*[a, e]

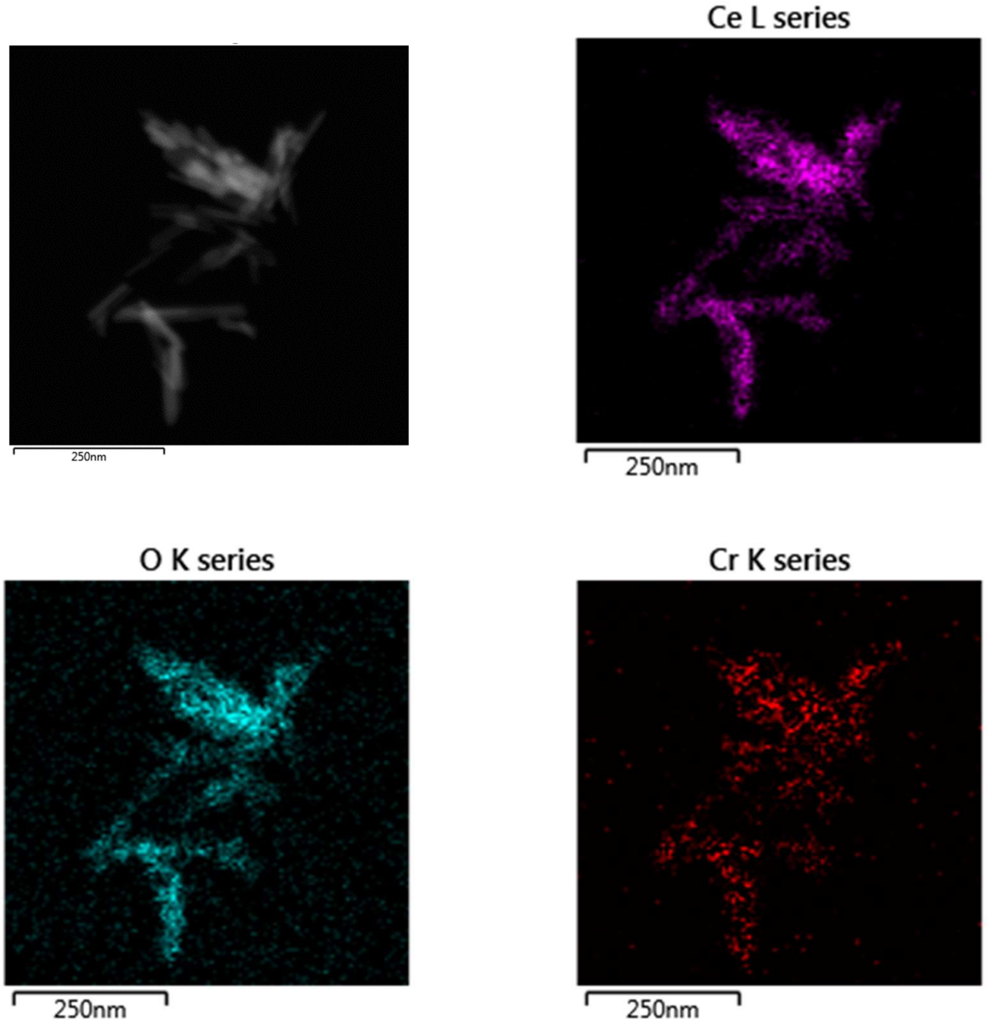

Figure S1. TEM-EDS analysis of 1%Cu-ceria nanorods.

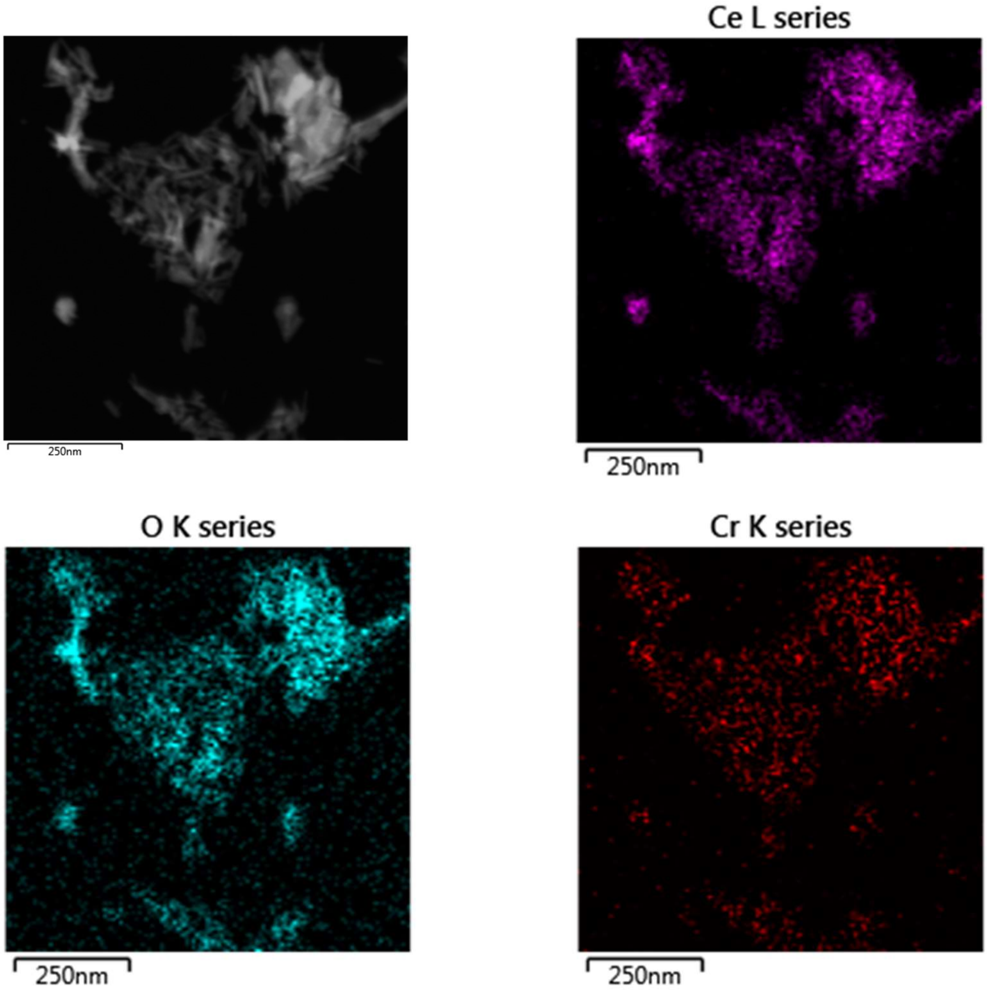

Figure S2. TEM-EDS analysis of 3%Cu-ceria nanorods.

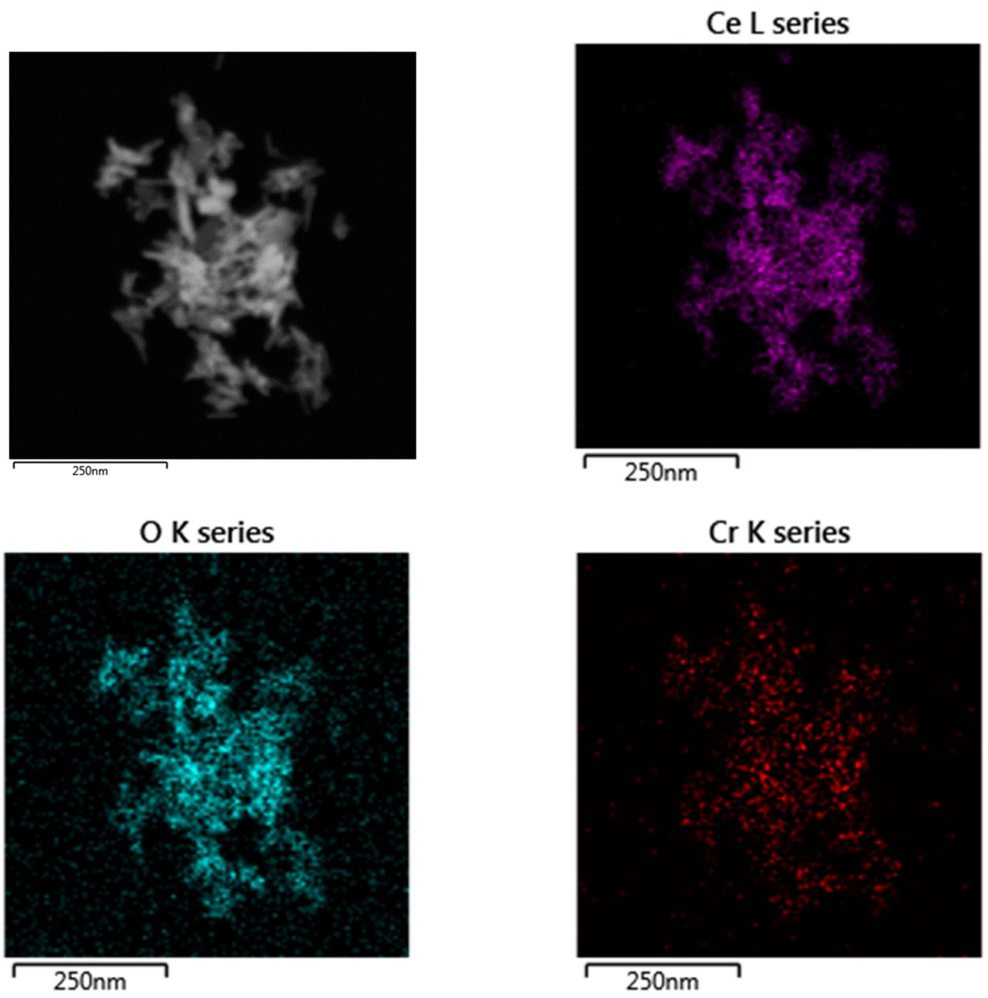

Figure S3. TEM-EDS analysis of 5%Cu-ceria nanorods.

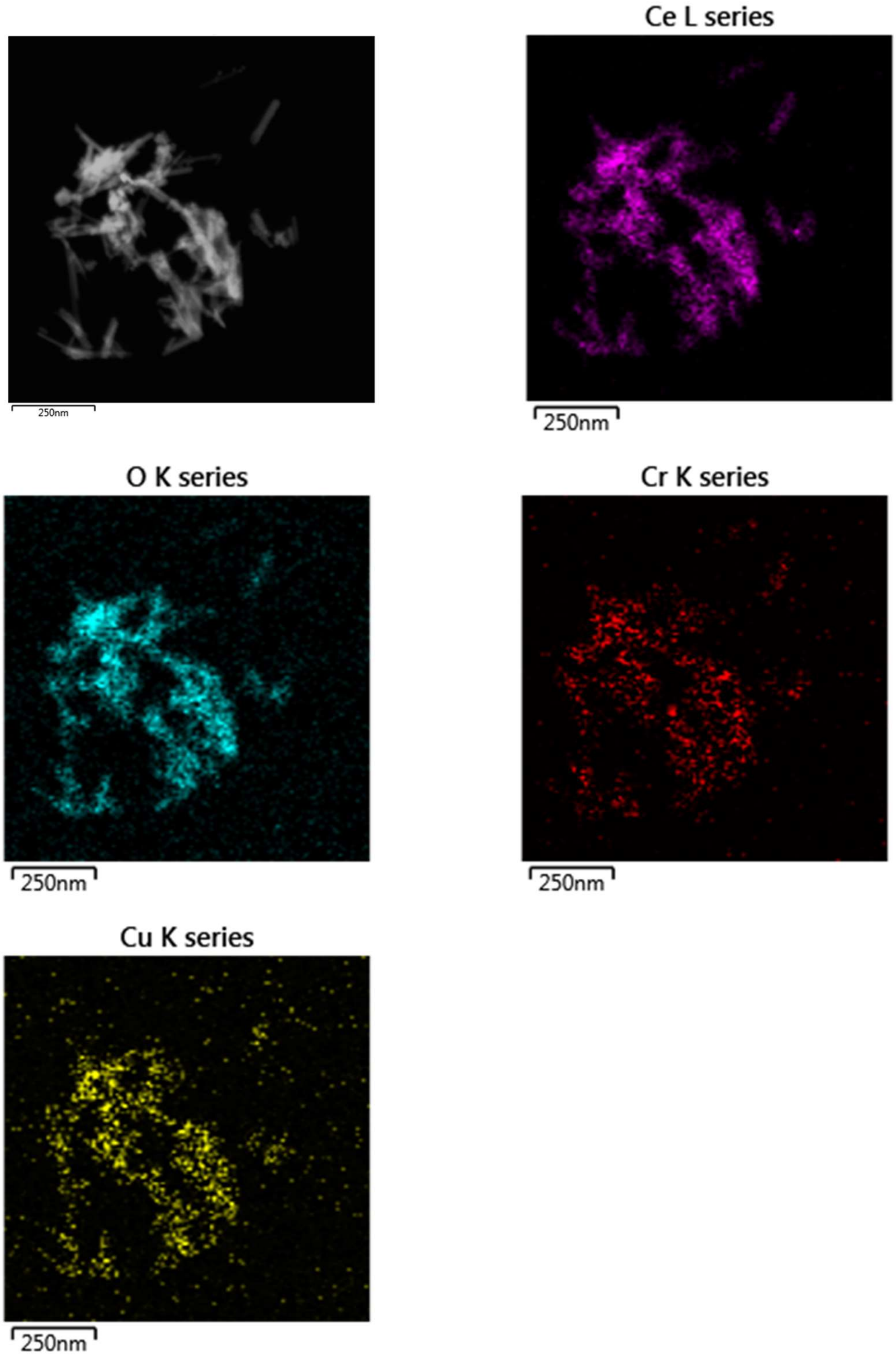

Figure S4. TEM-EDS analysis of 1%Cu/1%Cr-ceria nanorods.

Table S1. TPR peak locations and areas for undoped and doped ceria nanorods, using full Gaussian distributions as shown in Figure S1 for an example.

Peak	Centre (°C)	H_2 consumption [cm^3 (STP) H_2 g_{cat}^{-1}]
**Undoped ceria nanorods**		
Low temperature 1	359	7.6
Low temperature 2	467	1.9
High temperature 1	833	20.2
**1 wt. % Cu**		
Low temperature 1	333	1.7
Low temperature 2	421	5.0
High temperature 1	844	28.1
**3 wt. % Cu**		
Low temperature 1	297	0.9
Low temperature 2	367	5.6
High temperature 1	831	24.9
**5 wt. % Cu**		
Low temperature 1	275	1.1
Low temperature 2	338	7.4
High temperature 1	863	26.8
**7 wt. % Cu**		
Low temperature 1	197	2.5
Low temperature 2	222	5.0
Low temperature 3	272	7.9
High temperature 1	852	23.6
**1 wt. % Cr**		
Low temperature 1	303	3.2
Low temperature 2	418	6.5
High temperature 1	822	13.9
**3 wt. % Cr**		
Low temperature 1	295	3.9
Low temperature 2	435	10.4
High temperature 1	826	16.8
**5 wt. % Cr**		
Low temperature 1	304	2.4
Low temperature 2	414	8.7
High temperature 1	822	14.6
**1 wt. % each Cu and Cr**		
Low temperature 1	192	3.5
Low temperature 2	313	1.3
Low temperature 3	404	7.1
High temperature 1	798	11.3

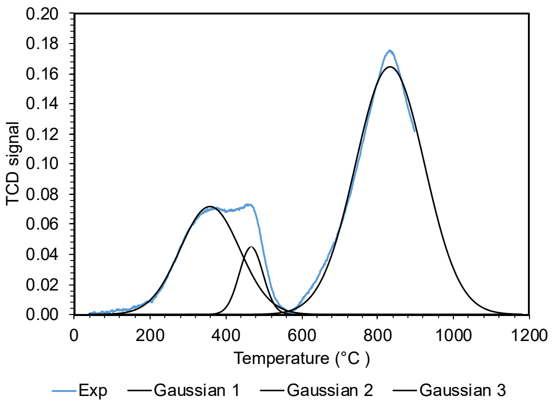

Figure S5. Deconvolution of the TPR experimental data with multiple Gaussian distributions (here for undoped CeO_2). The full area of each gaussian peak was accounted for the calculation of the hydrogen consumption in cm^3 (STP) H_2 g_{cat}^{-1}.

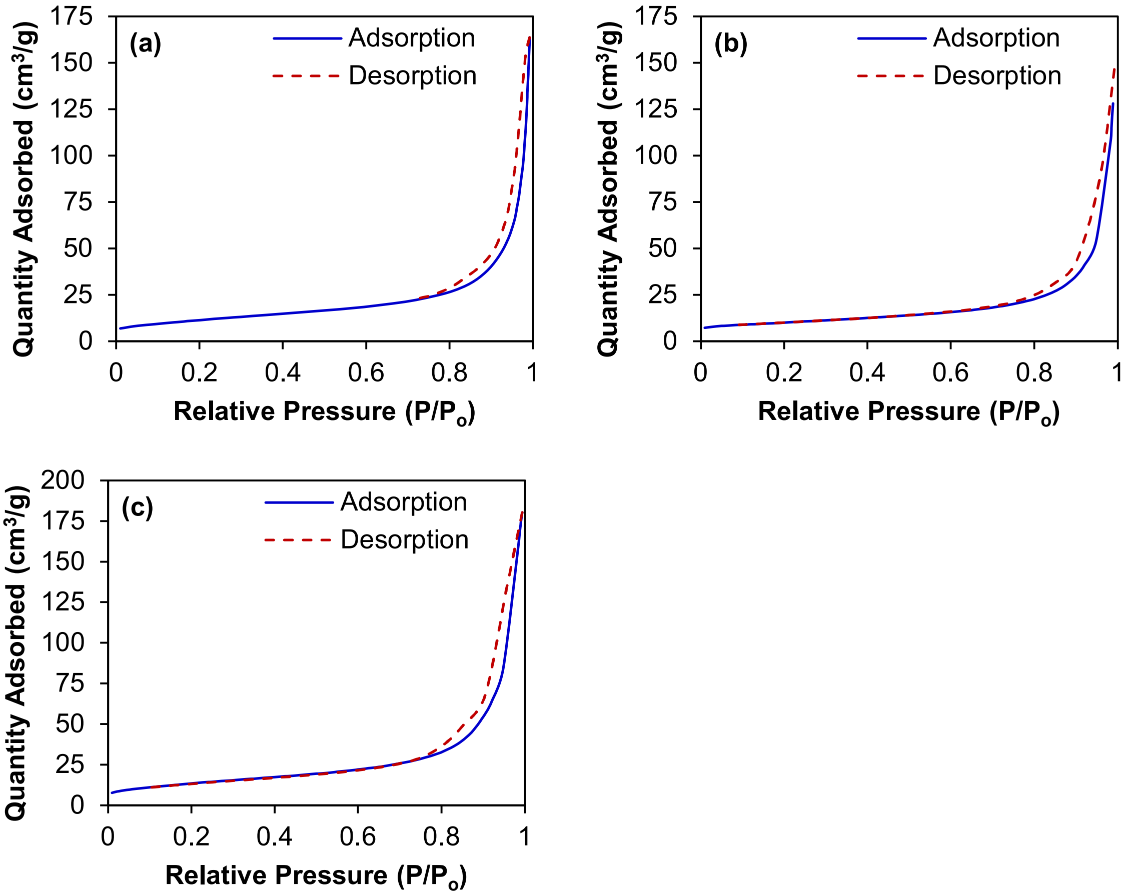

Figure S6. Nitrogen isotherms for (a) undoped ceria nanorods, (b) 1 wt. % Cu-doped ceria nanorods, and (c) 1 wt. % Cr-doped ceria nanorods.

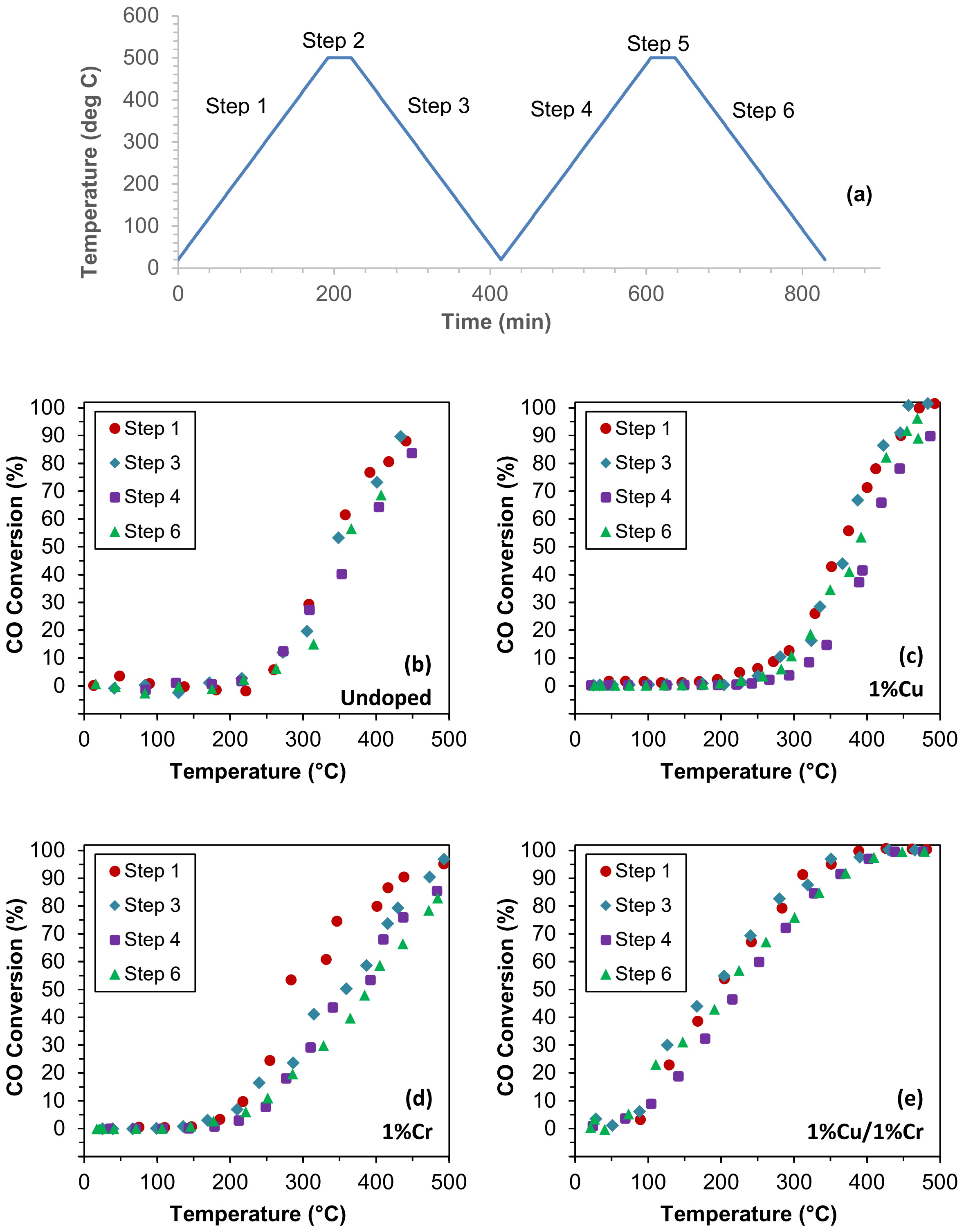

Figure S7. Repeated CO oxidation steps (a) at different temperatures for (b) undoped ceria nanorods, (c) 1 wt. % Cu-doped ceria nanorods, (d) 1 wt. % Cr-doped ceria nanorods, and (e) 1%Cu/1%Cr-ceria.

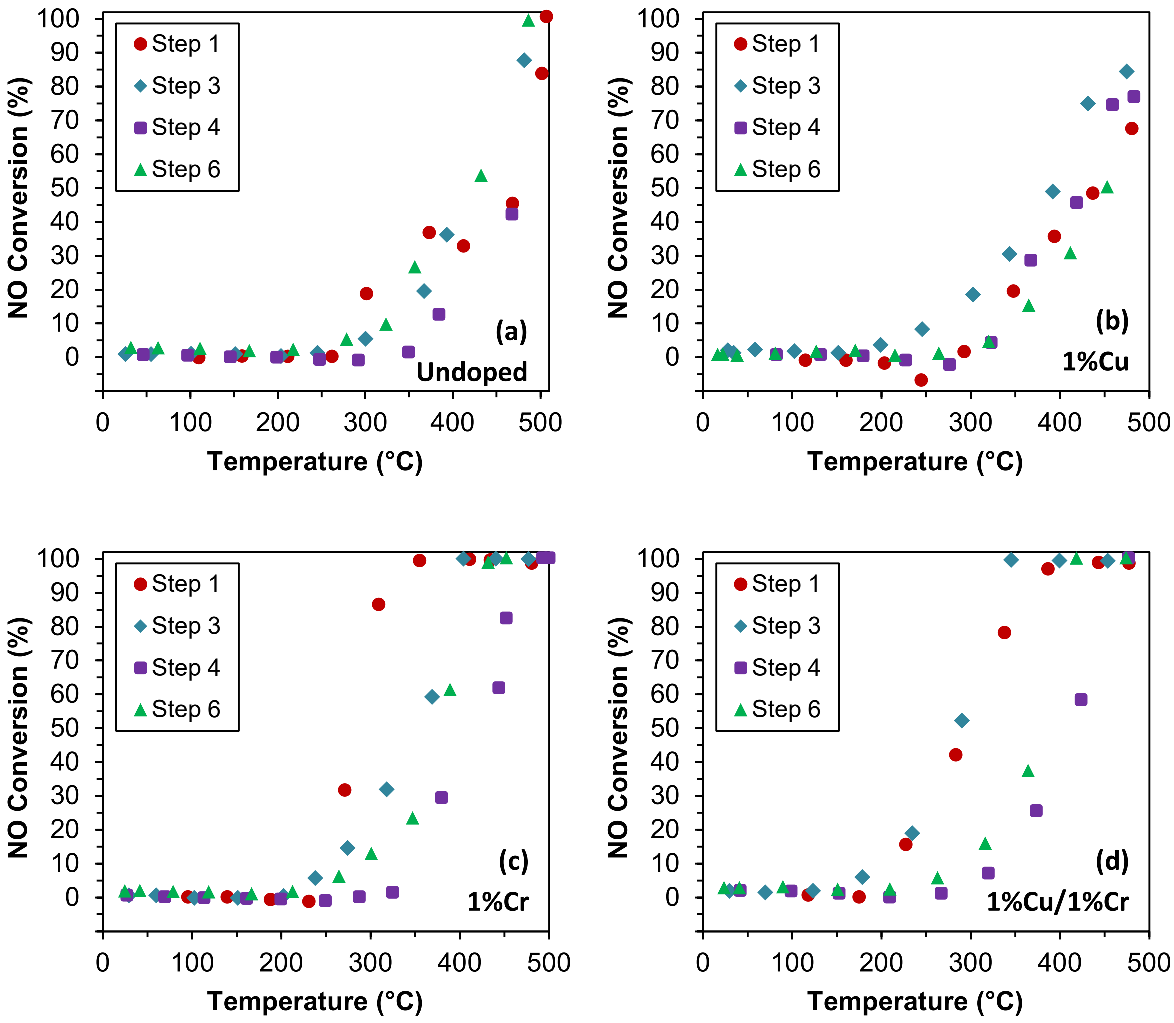

Figure S8. Repeated NO reduction steps for (a) undoped ceria nanorods, (b) 1 wt. % Cu-doped ceria nanorods, (c) 1 wt. % Cr-doped ceria nanorods, and (d) 1%Cu/1%Cr-ceria.

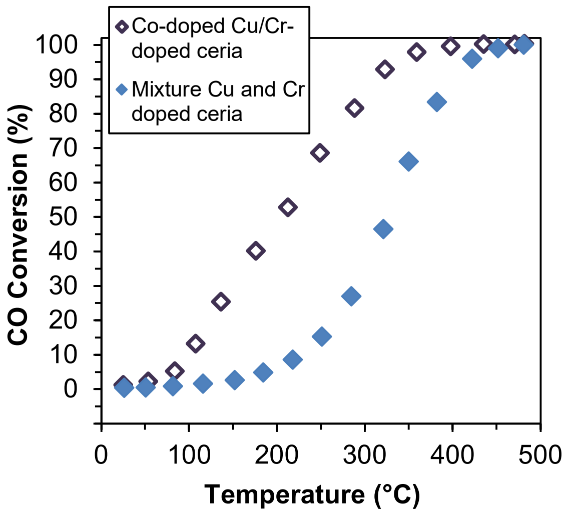

Figure S9. CO oxidation catalytic conversions for ceria nanorods co-doped with 1 wt. % each Cu and Cr (15 mg catalyst weight), compared with a physical mixture of 2 wt. % Cu-doped ceria and 2 wt. % Cr-doped ceria (7.5 mg each, to achieve equal amounts of Ce, Cr, and Cu).
